# Birth trauma in a population requiring inpatient mental health care in the postpartum period

**DOI:** 10.1177/10398562241246150

**Published:** 2024-04-14

**Authors:** Sophie Isobel, Alexandra Emerton, Sylvia Lim-Gibson

**Affiliations:** Naamuru Parent and Baby Unit, 222415 Sydney Local Health District, Camperdown, NSW, Australia; and; Faculty of Medicine and Health, 4334 University of Sydney, Camperdown, NSW, Australia; Naamuru Parent and Baby Unit, 222415 Sydney Local Health District, Camperdown, NSW, Australia; Naamuru Parent and Baby Unit, 222415 Sydney Local Health District, Camperdown, NSW, Australia

**Keywords:** mother baby unit, parent baby unit, perinatal mental health, birth trauma, post traumatic stress disorder

## Abstract

**Objective:**

This study explores rates of birth-related symptoms of trauma in a population of parents experiencing severe perinatal mental illness.

**Method:**

Birthing-parents admitted to a perinatal inpatient unit completed birth trauma measures on admission which were descriptively analyzed.

**Results:**

The population had higher rates of birth-related potentially traumatic events and trauma-related symptoms than the general population.

**Conclusions:**

The findings highlight that assessing for and responding to experiences of birth trauma is highly relevant to an inpatient perinatal population.

## Background

Birth trauma refers to experiences of interactions or events related to childbirth that cause overwhelming distressing emotions and reactions, leading to short or long-term negative impacts on health and wellbeing.^1^ All experiences of trauma are subjective, including birth trauma.^2^ However, events which may increase likelihood of births being considered traumatic include unexpected interventions, pain which is uncontrolled or beyond capacity to cope, possibility of injury or death to self or baby, or a sense that care providers are uncaring, unsafe or cruel.^3–5^ Like any other trauma, it is frequently the experience and effect not the events itself^6^ that comprise trauma, particularly feelings of powerlessness, fear, intrusion or betrayal, including the responses or actions of professionals.^7^ Many experiences of birth trauma are psychological and relational, embedded in interactions with care providers and dynamics of violation, betrayal or coercion.^8^ In mid-2023, Parliament in the state of New South Wales, Australia established a select committee to inquire into and report on birth trauma and related matters, with acknowledgment of high rates of birth trauma arising from inappropriate, disrespectful or abusive treatment before, during and after birth, also known as “obstetric violence.”^9^

Between 30%^10^ and 45%^11^ of birthing-parents identify their birth as “traumatic” without meeting diagnostic criteria for PTSD. The prevalence of PTSD related to birth at 6 weeks postpartum is thought to be 1.7%–6% of birthing-parents,^5^ with some rates as high as 4%–18.5%.^12^ However, PTSD is not routinely screened for across maternity and perinatal services.^13^ The main risk factors for postpartum PTSD are thought to be negative birth experiences, complications of birth or pregnancy, and a lack of professional or personal support,^13^ as well as a previous mental health diagnosis.^14^ Birth trauma is correlated to bonding difficulties, insecure attachment and parenting stress.^15^ Evidence suggests that birthing-parents who have experienced childbirth as traumatic may have difficulty bonding with their infants,^16,17^ less secure attachment towards their infant,^18^ and perceive the parenting role as more stressful at 2 years postpartum.^19^ Postnatal PTSD is also frequently comorbid with postnatal depression.^13,18^

The City Birth Trauma Scale (City-BiTS) was developed to identify birthing-parents at risk of developing postpartum PTSD.^20^ The 29-item self-report questionnaire follows the DSM-5 criteria of intrusions, avoidance, negative mood and cognitions, and hyperarousal.^15^ The City BiTS has a two factor structure-birth related symptoms and general symptoms.^15,20^ City-BiTS has been shown to be valid, reliable and clinically useful in the Australian context.^15,21^ The birth related symptoms subscale in particular is useful in delineating birth related PTSD from perinatal depression.^15^ Identifying birth related PTSD in clinical perinatal settings has a broad range of research and clinical applications.^15^

Inpatient Parent or Mother Baby Units (MBUs) provide acute mental health care to birthing parents experiencing moderate to severe perinatal mental illness. While units have been well established across Australia and the world,^22^ the first public MBU for New South Wales only opened in mid-2022. Included in the clinical measures introduced was the CITY-BiTS. To the best of our knowledge, there are no other studies which have examined the rates of birth trauma amongst the population admitted to an MBU.

## Aim

To explore rates of birth related symptoms of trauma in a population of birthing-parents experiencing severe perinatal mental illness.

## Method

From October 2022 to October 2023, 65 birthing-parents were admitted to the unit. At the time, the unit was the only public perinatal mental health unit in the state. As part of admission, parents are invited to complete questionnaires which are used to inform care-planning. Questionnaires include the Postnatal Risk Questionnaire (PNRQ)^23^; and the CityBiTS(20). During the study period, 43 birthing-parents completed admission questionnaires. Those with missing data were excluded leaving 37 completed PNRQ and CityBiTS responses, representing 57% of the population of parents in NSW requiring inpatient perinatal mental health care during the study period.

The PNRQ asks about risks and vulnerabilities for postnatal distress and includes a question about whether the experience of giving birth was disappointing or frightening. CITY-BiTS asks about birth-related experiences and associated symptoms of PTSD.

The study had ethics approval from the Hospital Ethics Committee.

## Findings

Total Birth related PTSD Symptoms scores ranged from 0 to 28 out of 30 with a mean of 5.32, median 2, st dev 7.18. See Tables 1 and 2.

**Table 1.** PNRQ Birth trauma endorsement

*N*37	%(n) endorsed
Was your experience of giving birth to this baby disappointing or frightening?	54% (20)

**Table 2.** CityBITS Birth trauma subscale

N37	%(n) endorsed
Did you believe you or your baby would be seriously injured during birth?	18.9% (7)
Did you believe you or your baby would die during birth?	18.9% (7)
In the past week have you experienced	
Recurrent unwanted memories of the birth (or parts of the birth) that you can’t control	29% (11)
Bad dreams or nightmares about the birth (or related to the birth)	11% (4)
Flashbacks to the birth and/or reliving the experience	31% (11)
Getting upset when reminded of the birth	38% (14)
Feeling tense or anxious when reminded of the birth	46% (17)
Trying to avoid thinking about the birth	30% (11)
Trying to avoid things that remind me of the birth	17% (7)
Not able to remember details of the birth	23% (8)
Blaming myself or others for what happened during the birth	27% (10)
Feeling strong negative emotions about the birth (e.g., fear, anger, shame)	32% (12)
If you have any of these symptoms (N23)	
Do these symptoms cause you a lot of distress?	96% (22)
Do they prevent you doing things you usually do?	83% (19)

## Discussion

This study reports on birth-related trauma in an inpatient perinatal population admitted to a Parent/Mother Baby Unit (MBU). The cohort was experiencing severe mental illness. Across the study cohort, potentially traumatic birth experiences were present for a high percentage of the population, varying from 18.9% (thinking they or their baby might die) to 54% (finding birth disappointing or frightening). This is higher than rates reported elsewhere. In an Australian community cohort study, for example, 14.4% of women thought they or their baby might die.^20^ Between 11 and 31% of the current study population experienced intrusions, 17%–30% experienced avoidance, and 27%–32% experienced negative cognitions and for the vast majority of women, these symptoms caused high levels of distress and impeded functioning, suggesting they may meet criteria for a diagnosis of birth-related PTSD. Rates of distress and functional impairment are similar to those reported elsewhere.^15^ Dissociative symptoms were less commonly reported amongst the cohort but still notable. Dissociative symptoms and emotional numbing have been previously correlated to parenting stress.^20^

A clinical cut-off for birth-related PTSD requiring intervention has not been established for the CITY-BiTS,^24^ however, the current study population are already experiencing psychological distress to a level requiring intensive clinical care. Subsequently, beyond diagnostic criteria, sub-threshold presentations of birth-related trauma are likely important as they are associated with impairment in psychological functioning in the clinical range.^10^ The high percentage of the study population who reported frightening or disappointing birth experiences therefore has clinical implications for responding to perinatal mental illness. The findings highlight that assessing for experiences of birth trauma is highly relevant to an inpatient perinatal population. Frequently, traumatic birth events are cumulative (e.g., a series of events that led to a perceived loss of control) or triggering of past trauma (e.g., intrusive internal exams).^10^ The subjective experience of events is crucial to understanding experiences of birth trauma, as two people can experience the same events in different ways^3,10^ and as such screening should be supported by clinical engagement and assessment. Birth-related trauma can differ from other forms of trauma in that it is often interwoven with conflicting emotions and physical, hormonal and psychological shifts.^18^ Unlike many traumatic events, birth is often anticipated to be a positive event, resulting in inner conflict about any associated negative cognitions.^15^

Unplanned interventions during birth are known to increase the risk of birth-related PTSD(15). Rising rates of interventional births are observed in Australia, despite relatively unchanged rates of maternal and infant deaths during childbirth,^15^ suggesting interventions may be being over-used. Parents with pre-existing serious mental illness are known to have higher rates of intervention during birth than parents without mental illness birthing in the same hospitals,^25^ although this relationship may undoubtedly be complicated. Yet, most birthing-parents report wanting to give birth with minimum intervention, and parents with mental illness are no different in their desires.^25^ Within the current study cohort 54% had delivered via caesarean section (28% in an emergency), which is significantly higher than the general population.^15^

The findings suggest that there is a need for routine assessment of birth-related trauma in perinatal mental health services. Rather than informing prevention of mental illness, as is the case in community health and maternity services,^26^ in mental health settings, information about birth-related experiences can directly inform formulations and care-planning. Clinical differences have been observed between people with birth-related PTSD and those who have co-morbid perinatal depression, with co-morbidity increasing symptom severity and functional impairment^27^ and the overlap between the two disorders presenting a clinically unique situation requiring targeted intervention.^15^ Approximately 40% of parents admitted to the MBU have a primary diagnosis of perinatal depression, suggesting a likely group for whom screening for birth-related trauma is essential.

A final reason for increased attention on birth-related trauma in the perinatal mental health context is the possibility for posttraumatic growth. Post-traumatic growth refers to positive changes in self-perception, philosophy of life and relationships, which can occur after enduring trauma-related difficulties.^28^ Post-traumatic growth following birth trauma is possible when parents are supported to engage in meaning-making and treatment.^11^ Thus, without acknowledging and responding to birth trauma, services may risk replicating traumatic dynamics and aggravating negative cognitions related to birth, self and baby, while missing opportunities to support wellbeing and recovery.

## Limitations

The findings of this study are descriptive only and form an exploratory ‘first step’ in considering the relevance of birth-related trauma to an acute mental health setting using a small population. In addition, this study only gathered data on the experiences of birthing parents, yet there is increasing understanding that non-birthing parents may also experience trauma from observing and witnessing events.

## Conclusion

This study identified high rates of birth-related symptoms of trauma in a population of parents experiencing severe perinatal mental illness and provides indication of a need for further research and consideration of the clinical implications for care delivery in perinatal mental health services. 
